# Spinal Arachnoiditis Ossificans: A Case-Based Update

**DOI:** 10.1055/s-0041-1731448

**Published:** 2021-07-22

**Authors:** Anna Brunner, Marlene Leoni, Sandro Eustacchio, Senta Kurschel-Lackner

**Affiliations:** 1 Department of Neurosurgery, Medical University Graz, Graz, Austria; 2 Institute of Pathology, Medical University of Graz, Graz, Austria

**Keywords:** arachnoiditis ossificans, spinal surgery, yellow bone marrow, cauda equina syndrome

## Abstract

Arachnoiditis ossificans is a rare disease, characterized by intradural ossifications, representing the end stage of chronic adhesive arachnoiditis. We describe the case of a 55-year-old patient who developed symptoms of a cauda equina syndrome after an open microdiscectomy at the L5 to S1 segment. A subsequent exploratory surgery revealed an intradural concentric bony structure with partly incorporated and partly adherent nerve roots. A partial removal of the intradural calcifications was performed. Postoperatively, the patient showed neurological improvement. The removed intradural calcifications were submitted for histological analysis and proved to be normal bone tissue, notably containing yellow bone marrow. To our knowledge, the presence of yellow bone marrow within bony cavities of arachnoiditis ossificans has not previously been reported.

## Case Report

### Clinical Presentation

A 55-year-old man presented with a 1-month history of low back pain and a left-sided weak plantarflexion. The Lasègue's sign was positive on the left side and a hyperactive patellar reflex on the left side was detected. There was no history of trauma, previous spine operations, or intrathecal injections. His clinical history revealed an episode of low back pain and sphincter disturbances 16 years ago.

**Fig. 1:**
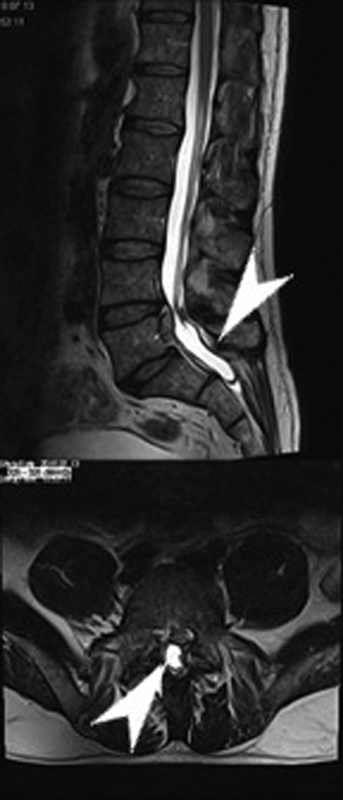
A sagittal and axial T2-weighted magnetic resonance imaging obtained before admission demonstrating a left-sided herniated disc at the L5 to S1 segment (arrowheads).

The initial spinal lumbar magnetic resonance imaging (MRI) showed a left-sided herniated disc at the L5 to S1 segment ( Fig. 1 ).

Due to the failure of nonsurgical management, the patient was submitted to an open microdiscectomy. The surgical intervention was uneventful; however, postoperatively the patient developed symptoms of a cauda equina syndrome with saddle anesthesia and bladder dysfunction (urinary retention).

### Imaging Findings

**Fig. 2:**
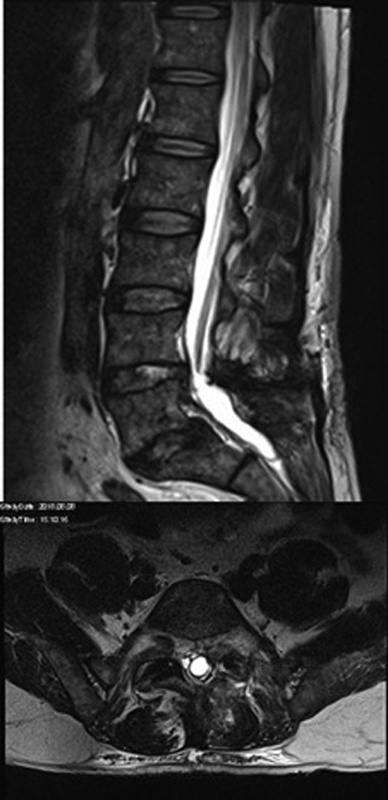
A T2-weighted magnetic resonance imaging (sagittal and axial view) performed after initial surgery showing a cystic enlargement of the dural sac from L5 to S2.

A spinal MRI was performed immediately and demonstrated a cystic enlargement of the dural sac from L5 to S2 ( Fig. 2 ).

### Surgical Intervention

**Fig. 3:**
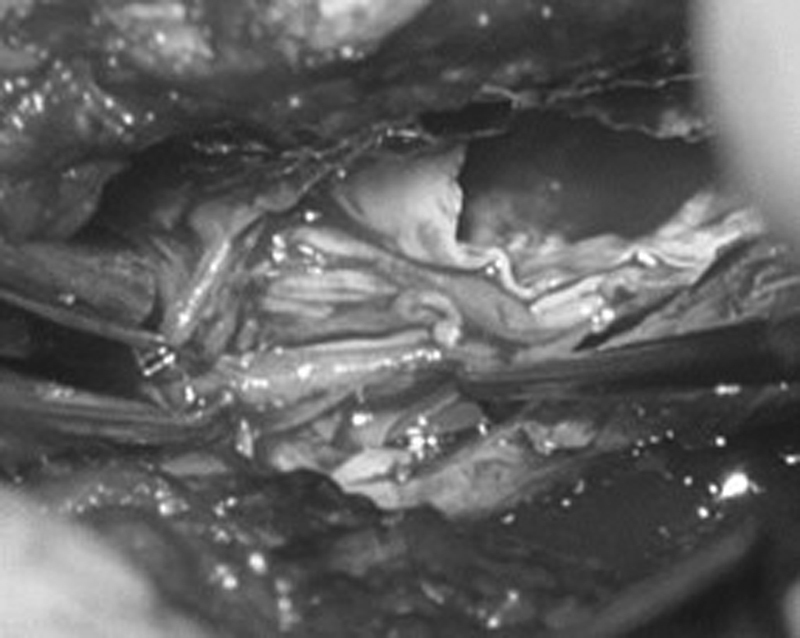
Intraoperative photograph demonstrating a concentric bony structure and tethered nerve roots.

An exploratory spinal surgery was performed and the dural sac at L5 to S1 was exposed. An extremely hard dural sac was detected, leading to the decision of a durotomy. A concentric bony structure was discovered in the dural sac. Nerve roots were partly incorporated by calcifications and partly adherent to the wall of this concentric bony structure ( Fig. 3 ). A partial removal of the intradural calcifications was performed and the nerve roots were separated with care.

### Histological Findings

**Fig. 4:**
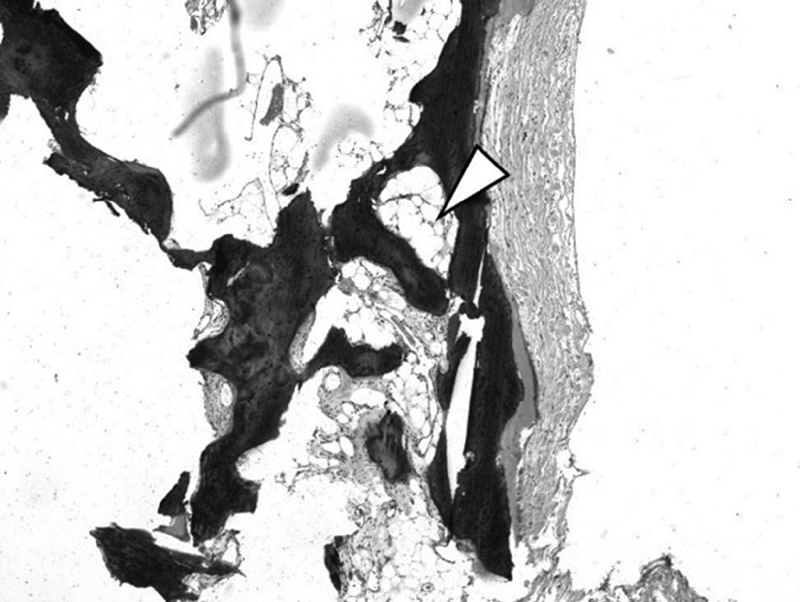
Photomicrograph of the intradural specimen showing yellow bone marrow within the intradural bone (arrowhead).

The removed intradural calcifications were submitted for histologic examination and revealed normal osseous tissue. Yellow bone marrow was found in the bony cavities ( Fig. 4 ).

### Postoperative Course

Postoperatively, the patient showed neurological improvement; the saddle anesthesia regressed, the bladder function turned normal, and the strength of the left plantarflexion improved.

**Fig. 5:**
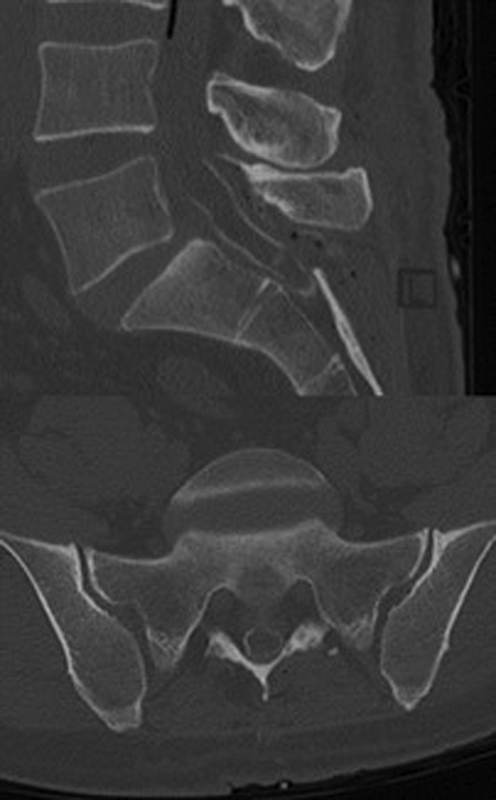
A bone window setting computed tomography (sagittal and axial view) performed postoperatively demonstrating the full extent of the remaining intradural bone formation.

A computed tomography (CT) was subsequently performed and demonstrated the extent of the intraoperatively detected intradural ossification ( Fig. 5 ).

The findings were consistent with spinal arachnoiditis ossificans.

## Review of the Literature

Arachnoiditis ossificans has to be distinguished from common benign meningeal ossifications. 1 Benign meningeal ossifications are frequently seen with an incidence up to 76% in autopsy studies and regarded as secondary age-related degenerative processes or as disturbed calcium metabolism. 2 3 These leptomeningeal calcifications are generally asymptomatic and have no clinical significance. 4 The differentiation of arachnoiditis ossificans from benign leptomeningeal ossifications was determined by Kaufmann and Dunsmore in 1971. 5 6 Histologically, arachnoiditis ossificans is distinguished from benign leptomeningeal ossifications, which displays with calcium crystals, by the detection of bone formation. 5 6

Arachnoiditis ossificans is most frequently located in the thoracic spine (66%); this is explained by the highest concentration of arachnoid cells in these segments. 4 5 7 The next common site is the lumbosacral region (24%). Only a few case reports describe the cervical spine as the site of occurrence. 3

The disease has shown to be associated with infective, traumatic, iatrogenic, or vascular events. 1 5 A history of intradural surgeries, myelographies, spinal anesthesia, or subarachnoid hemorrhages has been described in case reports. 7 8

The mean age at diagnosis is 53 years and there is no gender predilection. 3 5

## Clinical Features

Given that arachnoiditis ossificans mainly occurs at the thoracic and lumbar level, symptoms typically consist of longstanding backpain and radiculopathy proceeding to progressive myelopathy. 5 Symptoms and signs include (para)paresis, sensory disturbances, spastic gait, and urinary dysfunction. 1 2 5 7 9

## Radiological Features

In the absence of CT scanners, only severe forms of arachnoiditis ossificans have been detected by plain X-rays of the spine. 8 In this case, the disease has been found accidentally via an exploratory durotomy in the presence of neurologic deficits.

Nowadays, arachnoiditis ossificans can be reliably diagnosed by MRI or CT. On MRI, clumped nerve roots refer typically to a spinal arachnoiditis. Intradural ossifications appear hyperintense in T1-weighted images and may be hypo- or hyperintense in T2-weighted imaging. 5 8 MRI is helpful in displaying the involvement of nerval structures and for evaluation of potentially associated conditions as syringomyelia or arachnoid cysts. 2 5 Detection of bony structures and confirmation of the diagnosis is best achieved by CT. 5 8 Domenicucci et al classified the disease based on the ossification pattern on CT scans and described three types: in type I a semicircular ossification occurs, type II consists of a circular ossification and in type III a honeycomb like ossification is seen involving the entire contents of the dural sac. Type I is typically found in the thoracic region, type II occurs in the thoracic and lumbar region, and type III occurs only in the lumbar region, mostly with involvement of the cauda equina. 10

## Pathogenesis

The pathogenesis of arachnoiditis ossificans is not yet sufficiently resolved. It is suggested that inflammatory processes occur, similar to chronic arachnoid inflammations of other causes. 4 5 These processes are supposed to result in an uncontrolled proliferation of arachnoid cells with concomitant overproduction of collagen tissue and intradural scarring. 3 4 7 Subsequently, an increase in osteoblastic proliferation and bony metaplasia occurs, leading to progressive ossification. 3 6 8 Systemic metabolic conditions, particularly alterations in calcium metabolism, like hyperparathyroidism, seem to predispose to the formation of arachnoiditis ossificans. 1 5

Arachnoiditis ossificans is affecting nerval structures (spinal cord, nerve roots) by inflammatory processes or direct mechanical impairment, leading subsequently to neurological deficits. Moreover, the circulation of the cerebrospinal fluid may be affected by space occupying intradural osseous structures that can result in the formation of syringomyelia and arachnoid cysts. 1 2 3 6

Spinal vascular abnormalities, such as vascular malformations, have been found in patients presenting with arachnoiditis ossificans. It is hypothesized that recurred bleeding generates inflammatory processes. 1 3 8

## Histological Findings

Pathohistological specimens of these intradural ossifications reveal normal bone tissue, trabecular or lamellar in structure. 2 5 9 Other histological findings include psammoma bodies and clusters of meningothelial proliferation. 5 Signs of inflammation, as leukocytes and typical inflammatory matrix have also been detected. 5

## Treatment

Due to the rarity of the disease, no general treatment recommendations exist.

The therapeutic strategy should be based on the clinical presentation, the ossification pattern, and associated conditions like syringomyelia. Surgical treatment should be reserved for patients in whom rapid neurological deterioration is noted. Goals of surgery include decompression of the affected neural structures (spinal cord, nerve roots) and reestablishment of a physiologic cerebrospinal fluid circulation. Therefore, decompression of the spinal canal over the entire length of the ossification, for example via a laminectomy, is recommended as the treatment of choice. 5 6 10

Removing calcified plaques adhering the spinal cord or the nerve roots should be avoided. Particularly in type III arachnoiditis ossificans, an aggressive removal of adherent osseous plaques from neural tissue can cause significant deterioration of neurological deficits. 10

A few authors propose the placement of syringopleural or ventriculoperitoneal shunts to normalize altered cerebrospinal fluid dynamics. 1 5

## Discussion

In our case, arachnoiditis ossificans was detected accidentally when performing an exploratory spinal surgery in a patient presenting with secondary deterioration after an uneventful lumbosacral microdiscectomy.

It is noticeable that the clinical history revealed no predisposing events disregarding a temporary episode of low back pain and sphincter disturbances more than a decade ago. These findings had not been further explored by imaging and disappeared spontaneously.

Retrospectively, the cystic intradural formation was clearly apparent on the spinal MRI on admission. Neither the radiological findings nor the surgeon performing the initial procedure paid attention to this alteration. No suspicious findings particularly with regard to nerval structures or the dural sac were described in the surgical report.

It can be speculated that the postoperative development of the cauda equina syndrome in our patient is most likely attributable to intraoperative manipulations of the dural sac and the osseous incorporated nerve roots while performing microdiscectomy.

However, in the knowledge of an arachnoiditis ossificans, a (hemi)laminectomy of L5 followed by the removal of the herniated lumbosacral disc may be regarded as an optimal surgical approach in this case. It has to be debated whether this approach would have sufficiently reduced surgical manipulations during microdiscectomy to prevent postoperative neurological deterioration.

Histological analysis revealed normal bone tissue with yellow bone marrow. This is the first report describing this finding in arachnoiditis ossificans. We suggest that the formation of yellow bone tissue is caused by an adipose metaplasia of the arachnoid.

## Conclusion

In conclusion, arachnoiditis ossificans is rare, but should be kept in mind, particularly in patients with a previous history of spinal interventions or trauma. In these patients, clumped nerve roots and signal alterations, suspicious of intradural ossifications on MRI, should be further evaluated by CT scan. If arachnoiditis ossificans diagnosed, surgery should be performed only in patients with neurological deterioration. The recommended procedure is a bony decompression of the affected nerval structures; durotomy and resection of osseous plaques should be avoided.
